# Peripheral PD-1^+^ T Cells Co-expressing Inhibitory Receptors Predict SVR With Ultra Short Duration DAA Therapy in HCV Infection

**DOI:** 10.3389/fimmu.2019.01470

**Published:** 2019-06-27

**Authors:** Sara Romani, Kristen Stafford, Amy Nelson, Shashwatee Bagchi, Shyam Kottilil, Bhawna Poonia

**Affiliations:** Institute of Human Virology, University of Maryland School of Medicine, Baltimore, MD, United States

**Keywords:** chronic HCV, PD-1, relapse, 4 week DAA therapy, immune response, short-duration DAA, SVR

## Abstract

Direct acting antiviral (DAA) regimens of 12 weeks result in HCV clearance in vast majority of patients across genotypes. We previously demonstrated an ultra-short regimen of 4 weeks DAA cleared HCV in a subset of patients. Here, we hypothesized that individual level of antiviral immunity differentially influenced viral clearance and investigated biomarkers of a successful response. Cohorts of HCV patients treated for 4 weeks with DAA therapy who either achieved sustained virologic response (SVR) or relapsed were compared at baseline and at end of therapy (EOT) for immune cell phenotypes and HCV specific immunity. Higher levels of PD-1^+^ CD8^+^ and CD4^+^ T lymphocytes co-expressing inhibitory receptors (IR) were present at baseline and at EOT in HCV patients who eventually achieved SVR compared with those who relpased. HCV specific CD8^+^ T cells were predominantly contained within these IR expressing PD-1^+^ subsets. Patients in the SVR group had significantly higher CD8^+^ T cell degranulation in response to HCV peptides at baseline and higher levels of cytokine producing T cells at EOT time-point, relative to those who relapsed. In *ex vivo* cultures, PD-1^+^CD160^+^ CD8^+^ T cells had higher HCV specific degranulation and PD-1^+^2B4^+^ CD8^+^ T cells had higher cytokine expression (IFNγ^+^TNFα^+^ or IFNγ^+^CD107a^+^) compared with single or no IR expressing subsets, indicating higher virus specific functional capacity of these subsets. Receiver operating characteristics curve (ROC) for baseline circulating frequencies of PD-1^+^CD160^+^, PD-1^+^Tim-3^+^ CD8^+^ T cells and PD-1^+^CD160^+^, PD-1^+^Blimp-1^+^, PD-1^−^CTLA4^+^ CD4^+^ T cells respectively, had associated C-statistics of 0.8214 and 0.9451 for discriminatin of patients who successfully cleared HCV with 4 weeks treatment. Thus, PD-1^+^ virus-specific CD8^+^ T cell subsets with cytotoxic capacity are present in a subset of chronic HCV infected individuals that associate with ability to achieve SVR, indicating role of immunity in DAA mediated viral clearance with short duration therapy.

## Introduction

Chronic hepatitis C virus infection is a major disease worldwide with over 170 million infections and remains a leading cause of chronic liver disease. With the advent of direct acting agents (DAA), HCV cure now occurs in most patients treated with standard 12 weeks regimens. Outcomes with treatment for 8 or 6 weeks are similar to standard duration resulting in cure rates of >95% (1, 2). However, the response rates dropped to 30% when patients were treated for 4 weeks with DAA combinations (3). These results indicate that antiviral therapy alone is not sufficient to achieve cure or sustained virologic response (SVR) in most patients when they are treated for 4 weeks. In this regard, we investigated factors associated with response to short duration therapy and hypothesized that those who were able to achieve SVR likely had effective antiviral immunity that contributed to viral clearance. Defining biomarkers of successful response to short duration treatment can potentially increase the reach of these therapies by reducing cost-burden associated with them. Recent studies suggest there is potential role of host immunity in determining response to DAA therapy. Higher serum levels of type 1 or 2 cell associated cytokines/chemokines are present in patients with rapid virologic response (4). Similarly, frequencies of baseline CD3^+^ and naive CD8^+^ T lymphocytes reliably identified patients that achieve rapid reduction in viremia following DAA therapy (5). While these studies suggest host immunity affects response to DAA, rapid viral load decline does not lead to SVR and these immune biomarkers cannot be applied to selecting potential responders to short duration therapy. By evaluating immune responses of patients who achieved SVR or relapsed with 4 weeks therapy we expected to identify immune phenotypes that can predict clinically relevant response, SVR, to such regimens.

It is inferred from natural infection studies that host immunity plays a role in HCV clearance. In acute resolving infection, virus specific CD8^+^ T cells play critical antiviral function (6, 7). Chronic infection results in damaging this antiviral immune system, thus persistently infected patient is unable to mount an immune attack on infected cells. HCV chronicity results in reduced numbers of or impaired functional capacity of HCV-specific T cells. Prolonged or excessive stimulation of T cells with HCV antigens leads to progressive T-cell exhaustion characterized by expression of a number of inhibitory receptors, including PD-1 on virus-specific T cells (8, 9). In addition to this direct effect on HCV-specific T cells, chronic infection results in global immune dysregulation (10, 11). Treatment with DAA results in rapid reduction of HCV viremia and could release immune cells from viral particle mediated inhibition. Since chronically HCV-infected subjects have dysfunction of HCV specific immune cells, it is conceivable that suppression of HCV viremia will result in rejuvenated HCV specific immune response, which may enhance HCV clearance. Direct antiviral therapy mediated antigen removal results in reduced PD-1 expression on and improved proliferation of HCV specific CD8^+^ T cells (12). In patients who failed DAA therapy, re-treatment with same regimens resulted in high cure rates of >90% (AASLD 2018 presentation 0583; unpublished data). In these cases, it is plausible that patients recover some degree of immune function with the first treatment regimen and re-treatment provides more profound virus suppression that allows repair of anti-viral immunity to levels that overcome virus replication when therapy ends (13). We previously treated patients for 4 weeks with DAA regimens where all treated patients achieved a reduction in viral load to below detectable levels within 4 weeks of treatment initiation and 30% of those succeeded in achieving SVR (3). The main objectives of the present study were to investigate role of host immunity in achieving SVR with short term DAA therapy and to derive prediction tool based on immune markers that would identify responders to such regimens. We show that sustained virologic response associates with host immunity. Higher HCV specific CD8^+^ T cell response and circulating PD-1^+^CD8^+^ or CD4^+^ T cell subset frequencies at baseline were identified as potential biomarkers of successful response to 4 weeks DAA therapy in ~90% patients.

## Materials and Methods

### Subjects and Samples

Treatment-naïve subjects who attended a single site, the National Institutes of Health (NIH) Clinical Center in Bethesda, Maryland during January 2014 to May 2015 were enrolled in this study. Patients were aged 18 years or older and had chronic HCV genotype 1 infection (serum HCV RNA level ≥2,000 IU/mL) and were treated with LDV/SOF/GS-9451 (Vedroprevir) and LDV/SOF/GS-9451/GS-9669 (Radalbuvir) regimens for 4 weeks. Full eligibility criteria and results of the trial are described elsewhere (Clinical Trials.gov: NCT01805882) (3). In all 30% of patients achieved primary endpoint of SVR12 (HCV RNA levels below the lower limit of quantification posttreatment week 12); rest of the patients had virological relapse, with HCV RNA reappearing after stopping therapy. Based on availability of sufficient number of cells, we selected *N* = 13 from “SVR” and *N* = 13 from “relapse” groups for the present investigation. Detailed demographic and clinical characteristics of the 26 patients from whom samples were utilized in the present study are provided in Table 1. treated with a standard 12-week DAA regimen (sofosbuvir, velpatasvir, and voxilaprevir) were available. Another cohort comprised of race and age matched healthy blood donors (*N* = 12) and served as HCV negative controls. All patient samples used are from already existing collections. All participants signed informed consent approved by the National Institute of Allergy and Infectious Disease Institutional Review Board at the time of screening and enrollment and all samples were anonymized. All methods utilized for this study were performed in accordance with the relevant guidelines and regulations.

**Table 1:** Demographic and clinical characteristics of relapse (*N* = 13) and SVR (*N* = 13) patients.

**ID**	**Genotype**	**Age Mean ± SD**	**Race**	Viral load^*^	**ALT d0**	**ALTwk4**	ALTwk16^***^	**ASTd0**	**ASTwk4**	ASTwk16^**^	**HAI fibrosis**
**Relapse**											
G01	1a	56(8.3)	Black	530,843	72	23	109	43	30	57	0
G04	1a		Black	1,013,053	39	25	41	34	28	31	1
G09	1a		Black	2,370,113	43	22	53	48	26	79	1
G10	1a		Black	164,246	49	12	55	54	19	49	1
G14	1a		Black	3,487,857	41	14	45	50	25	47	1
G17	1a		Black	3,902,745	47	17	87	37	20	65	1
G18	1a		Black	2,766,209	71	21	63	52	19	46	F1-F2
G21	1b		White		34	21	16	34	21	20	F1-F2
G23	1b		Black	7,447,943	51	25	42	40	26	44	1
G25	1a		White		40	21		30	21		0
H03	1a		Black	7,593,432	25	10	195	28	18	117	1
H17	1a		Black	538,545	22	15	22	28	26	33	1
H24	1a		Black	5,708,650	41	19	58	35	23	64	2
Mean				**3,229,421**	44.2	18.85	65.5	39.46	23.23	54.33	
**SVR**											
H05	1b	51.4(10.9)	White	2,015,029	42	14	15	35	17	20	1
H11	1b		Black	586,670	68	21	16	72	31	30	0
H16	1b		Black	150,227	155	21	25	102	28	52	1
G02	1a		White	617	72	43	27	46	29	30	0
G05	1a		Black	255,677	72	20	26	58	24	31	F1-F2
G06	1a		White	197,158	56	29		33	24		0
G07	1a		Black	135,871	84	30	29	57	29	26	F1-F2
G08	1b		Black	659,991	30	15	12	32	22	20	1
G15	1a		Black	418,063	82	25	17	57	31	22	F1-F2
G16	1b		Black	998,920	22	12	8	25	19	16	1
G19	1a		Black	984,984	39	12	11	56	19	19	F1
G20	1b		White	508,934	36	15	14	31	19	23	F0-F1
G22	1b		Multiple Race	3,957,962	57	22		30	17		F0-F1
Mean				**920,367**	62.9	21.46	18.18	48.77	23.77	26.27	

^*^Indicates parameters significantly different between Relapse and SVR groups as determined by Mann Whitney or t-test, depending on data distribution.

* p <0.05,

** p <0.01,

*** *p <0.001. Bold indicates the mean values of viral load for each group*.

### PBMC Isolation

Blood samples were collected before treatment (baseline), at the end of treatment (EOT) at week 4 and 12 weeks after end of treatment in heparinized tubes. PBMCs were isolated by Ficoll-Paque (Pharmacia) density centrifugation. Cells were counted by trypan blue exclusion and stored in freezing medium containing DMSO in liquid nitrogen until use. PBMCs were thawed in complete medium [RPMI 1640 with 10% fetal bovine serum, 1% penicillin- streptomycin (all Life Technologies, USA)] for all immunological assays.

### HCV Peptides and Tetramers

Genotype 1a or 1b HCV 15-mer to 18-mer peptides with 11 or 12 amino acid overlaps spanning the entire HCV polyprotein (peptide array, hepatitis C virus; BEI Resources, NIAID, NIH) were reconstituted in 5% sterile dimethyl sulfoxide and pooled. Peptides were aliquoted and stored at −80°C until use. HLA-A^*^02:01 restricted NS5B tetramer ALYDVVTKL-APC and NS3 tetramer CINGVCWTV-PE from MBL-International, Woburn, MA were used along with surface markers to determine phenotypes of HCV specific CD8^+^ T cells.

### Immunophenotyping

Multicolor flow cytometry analyses were performed on thawed PBMCs at baseline, EOT and 12 weeks after end of treatment. Phenotyping was performed to assess the expression of multiple T cell lineage, activation and inhibitory receptors by using the following monoclonal antibodies in two different panels containing: Fixable Aqua Live/dead (Invitrogen), anti-CD3 Alexa Fluor 700 (BD Biosciences), anti-CD4 APC-Cy7 (BD Biosciences), anti-CD8 Brilliant Violet 510 (Biolegend), anti-CD244 PercP-Cy5.5 (Biolegend), anti-CD39 FITC (Biolegend), anti-Tim-3 PE-CY7 (eBioscience), anti-PD-1 Brilliant Violet 421 (Biolegend), anti- Blimp-1 APC (eBioscience), anti-CCR7 FITC (Biolegend), anti-CD45RO APC-ef780 (Biolegend), anti-CD160 PE-Cy7 (Biolegend), anti-CTLA-4 PE-Cy5 (Biolegend). PBMCs were stained with antibodies against surface markers, followed by staining for intracellular markers. For transcription factor detection, intranuclear permeabilization was done with eBioscience Transcription Factor Fixation/Permeabilization concentrate and diluent solutions (Cat. No. 00-5521) according to the manufacturer's instructions. Antibodies for intranuclear transcription factors anti-Eomes-PE, anti-T-bet-BV421, and anti-BLIMP-1-APC were then added to the cells, incubated for an additional 30 min on ice, washed, and fixed in 1% paraformaldehyde.

For phenotyping of HCV specific T cells, HLA-A^*^02 positive patient samples (*N* = 5) were tested. Between 1 and 5 million cells were stained with viable dye followed by fluorochrome conjugated tetramer, along with antibodies to detect CD3 and CD8. Cells were acquired on BD FACS Aria flow cytometer and data were analyzed by FlowJo version 9.7.7 software (TreeStar, Inc). FlowJo data analysis was performed by a single investigator blind to sample identification.

### Functional Assays

For assessing HCV-specific CD8 T cell function in SVR and relapsed patients, baseline and EOT PBMC were tested. Cells were thawed and rested overnight to aid in recovery from freezing conditions. At least 1 million PBMC were tested in each condition: unstimulated (negative control) or HCV peptide stimulated (1 μg/ml each peptide). Cells were incubated with respective stimulations along with addition of 10 IU/ml IL-2 for 3 days. On day 4, cells were re-stimulated with HCV peptides for 18 h along with addition of BV650 anti-CD107a antibody and golgi transport blockers (Golgi Stop 0.7 μl/ml and Golgi Plug 1 μg/ml (BD Biosciences). Cells were stained for Live/dead stain and surface markers including HCV tetramers where applicable. Cytokine production was detected by staining with anti-IFNγ BV421, anti-TNFα PE and anti-IL-2 PerCPCy5.5 antibodies following intracellular staining fixation and permeabilization protocol (BD Biosciences) and flow cytometry. Samples were considered positive if the following criteria were met: the percentage of cytokine-positive cells was >2 times background (non-stimulated cells), the percentage of cytokine-positive cells was >0.05% after background subtraction, and the population of positive cells was larger than 10 cells.

### Statistical Analysis

Statistical analysis of phenotypic and functional markers among lymphocyte was performed on GraphPad Prism 6 software (GraphPad Prism Software, Inc.) and using SAS 9.4 (Cary, NC). Statistical test used is indicated in respective figure legend. A *p*-value <0.05 was considered significant. Mean values are used throughout the manuscript if not otherwise stated. To determine candidate markers for modeling to predict SVR, cellular markers were grouped into baseline, 4 week, and end of treatment timepoints. Generalized linear regression models, stratified by cell type (CD4 or CD8) using stepwise selection were used to identify the immunologic parameters for further investigation in predictive models. The significance level for both entry and exit into the model was 0.05. Candidate markers (for each time period separately) retained by the stepwise model were then entered into generalized linear models for a binary outcome with a random intercept for each patient allowing for the correlated nature of the data (multiple markers from the same patient) to estimate the predicted probability of having achieved SVR. We ran separate stepwise models for immunologic parameters from each time point to also determine if a time point in the course of treatment was more predictive of SVR than other time points. Candidate markers (for each time period separately) retained by the stepwise model were then entered into generalized linear models for a binary outcome with a random intercept for each patient allowing for the correlated nature of the data (multiple markers from the same patient) to estimate the predicted probability of having achieved SVR. The resulting predicted probabilities were then used to plot Receiver Operator Characteristic curves and a C-statistic was calculated to assess the combination of predictors ability to accurately discriminate between SVR and relapse patients. We ran separate stepwise models for immunologic parameters from each time point to also determine if a time point in the course of treatment was more predictive of SVR than other time points.

## Results

### Increased Frequencies of PD-1^+^ CD8^+^ T Cells Co-expressing Inhibitory Receptors Tim-3, CD160, 2B4, KLRG1 and Blimp-1 in Patients That Achieved SVR

**Figure 1:**
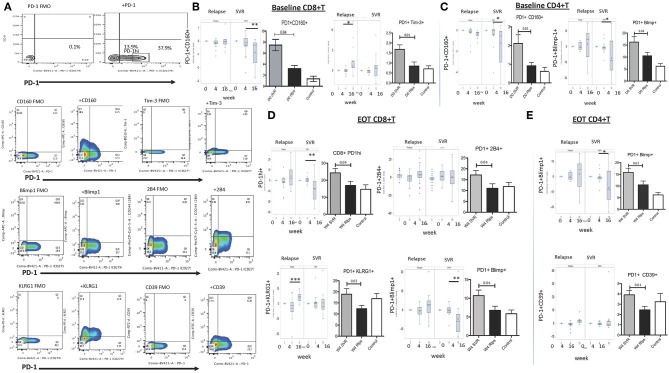
Expression of inhibitory receptors on global T lymphocytes in DAA treated SVR and Relapse groups. **(A)** Gating strategy for identifying T cells expressing PD-1 and other inhibitory receptors using appropriate FMO controls. For B-E, left panels in each figure show change in indicated subset's frequency relative to baseline (0) after treatment initiation (week 4 and 16) in relapse and SVR groups; right panels show grouped averages in SVR, relapse (Rlps) and healthy Controls. **(B)** Baseline (D0) PD-1+CD160+ and PD-1+Tim-3+ CD8+ T cells, **(C)** D0 PD-1+CD160+, PD-1+Blimp-1+ CD4+ T cells, **(D)** Week 4 (W4 or EOT) PD-1hi, PD-1+2B4+ PD-1+KLRG1+, and PD-1+Blimp-1+ CD8+ T cells and **(E)** Week 4 (W4 or EOT) PD-1+CD39+, PD-1+Blimp-1+ CD4+ T cells in SVR, relapse and healthy controls. *P-*values were determined by 1 Way ANOVA with Bonferroni's-Dunn's *post-hoc* test for pairwise multiple comparisons, *p* ≤ 0.05 is significant. Controls, healthy control; D0, baseline; W4, week 4/End of treatment (EOT); W16, 12 weeks after EOT. *p ≤ 0.05, **p ≤ 0.01, ***p ≤ 0.001.

Our objective was to find an immune phenotypic signature that can distinguish between SVR and relapse groups at baseline or at EOT. Since chronic HCV infection is characterized by global and antigen specific T cell dysfunction marked by exhaustion of T cells, we focused on phenotypic markers that identify various stages of T cell differentiation and exhaustion. Patients that achieved SVR had higher frequencies of central memory (CD45RO+CCR7+) CD4^+^ and CD8^+^ T lymphocytes at baseline and EOT timepoints than relapsers; the frequencies of effector memory (CD45RO+CCR7-) T lymphocytes were lower in SVR at baseline and increased significantly at week 16, while in relapsers effector memory frequency decreased significantly at week 16 (mean baseline, EOT, 16 week 20, 18, 40% SVR; 25, 24, 20% relapsers). This suggests an effect of SVR and absence of HCV replication on recovery of this population. Contrary to our expectation of a lower frequency of exhaustive phenotype in T cells from patients with SVR, we observed a signature of PD-1^+^ T cells co-expressing multiple inhibitory receptors (Figure 1; Supplementary Figure 1). Frequencies of the following PD-1 expressing subsets were significantly higher at baseline in SVR than relapse group: PD-1^+^Tim-3^+^ (mean 1.6,0.8), PD-1^+^CD160^+^ (mean 8.4,0.5.3), and at EOT: PD-1^hi^ (mean 24.26,16.9), PD-1^+^2B4^+^ (mean 17.16,11.02), PD-1^+^Blimp1^+^ (mean 10.7,6.7), PD-1+KLRG1+ (mean 18.9,12.3) (Figure 1). Similar results were obtained for CD4^+^ T cells with higher expression of PD-1^+^CD160^+^ (mean 2.1,0.9), PD-1^+^Blimp-1^+^ (mean 16.2,10.5) in SVR than relapsers. Other significant difference between the groups include higher frequencies of Eomes^+^PD-1^+^ CD8^+^ cells (mean 9,7, 6.5) and lower frequencies of CTLA-4 expressing PD-1^−^ CD4^+^ T cells in SVR relative to relapse at baseline (mean 4.8,7.6) (Supplementary Table 1). While higher frequencies of these PD-1^+^ subsets were maintained at week 4 (EOT) in SVR group compared to relapsed patients, at week 16 significant reduction was observed for PD-1^hi^, PD-1^+^CD160^+^ and PD-1^+^Blimp-1^+^ subsets relative to frequencies present at baseline and EOT in SVR group (Figures 1B–E). Meanwhile patients that relapsed had significantly increased PD-1^+^KLRG1^+^ and PD-1^+^Tim-3^+^ CD8^+^ T cells relative to their frequencies at baseline and EOT possibly in response to continuous antigen presence in this group.

### Immune Markers to Predict Responders to Short Duration Therapy

**Figure 2:**
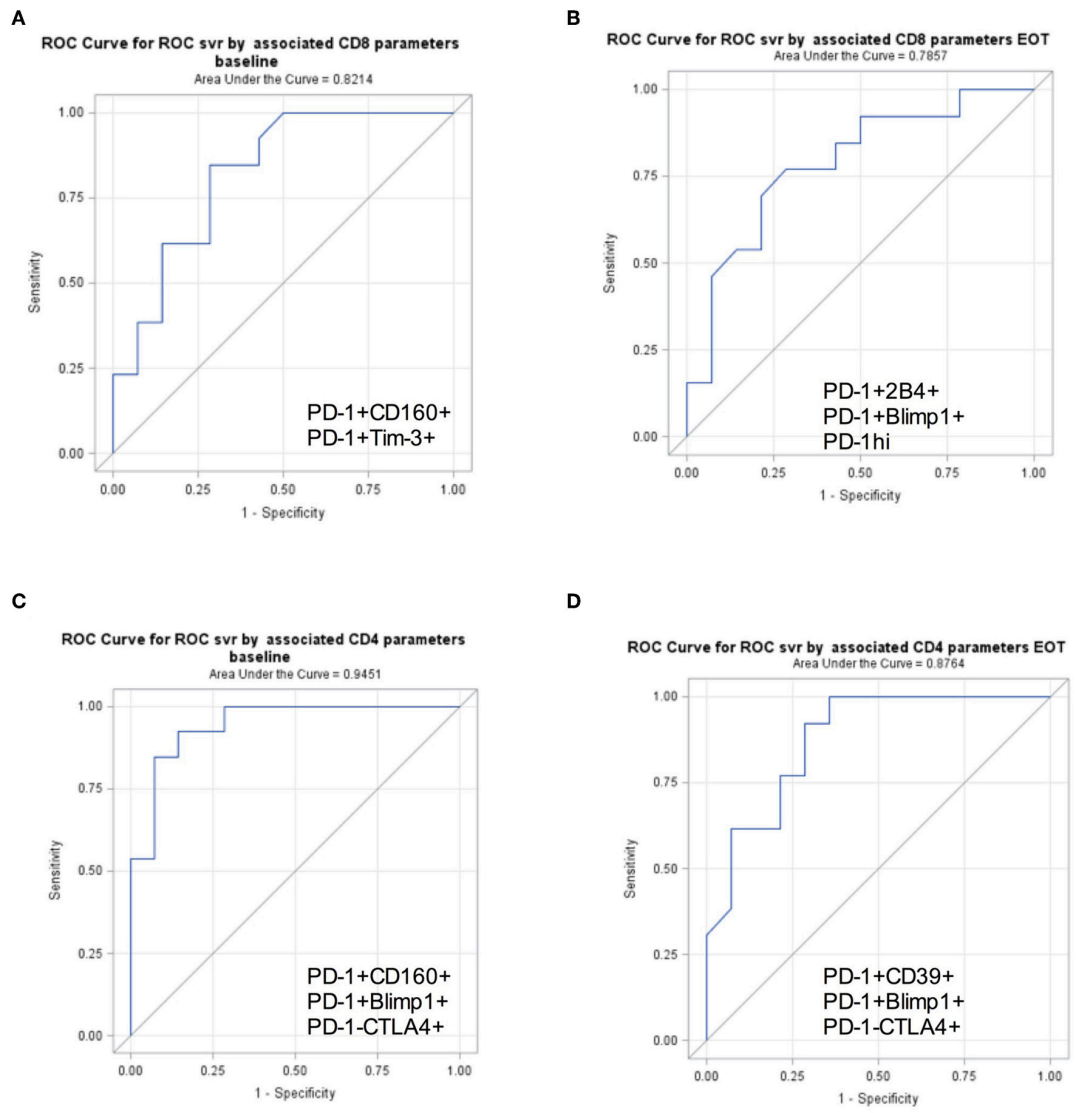
PD-1+ T cell subsets to predict SVR. Receiver Operator Characteristics curves for surface markers selected in stepwise generalized linear models for binary outcomes accounting for correlations within subjects demonstrating discrimination between SVR and relapse. Area under receiver operating characteristic curve (AUC) is shown for baseline and EOT CD8+ **(A,B)** and CD4+ **(C,D)** surface markers predicting SVR.

The ROC curves and the corresponding C statistics (equivalent to the AUCs) represent the percent of the time the model would correctly discriminate between patients who achieved SVR from patients who did not. Baseline CD4 parameters which provided the strongest discrimination between SVR and relapse were Blimp^+^PD1^+^, CD160^+^PD1^+^, and PD1^−^CTLA^+^ cellular expression levels (C-statistic = 0.94) (Figure 2). At end of treatment, CD4 Blimp+PD1^+^, PD1+CD39+, and PD1^−^CTLA4^+^ cellular expression levels provided the strongest discrimination (C-statistic = 0.87). Among CD8 parameters, at baseline PD1+Tim3+ and PD1+CD160+ provided the strongest discrimination (C-statistic = 0.82). At end of treatment CD8 PD1^hi^, PD1^+^2B4^+^, and Blimp^+^PD1^+^ provided the strongest discrimination between SVR and relapse (0.79). When trying to validate the components using PCA, none of the immune markers had an eigenvalue value of.7 or greater and thus PCA was not investigated further. Thus, higher baseline PD-1 co-expressing CD8^+^ and CD4^+^ T cell subsets could respectively, predict probability of achieving SVR in ~82 and 94% patients.

### Higher Frequencies of CD8^+^ T Cells With HCV Specific Functional Potential in Patients Who Achieved SVR

**Figure 3:**
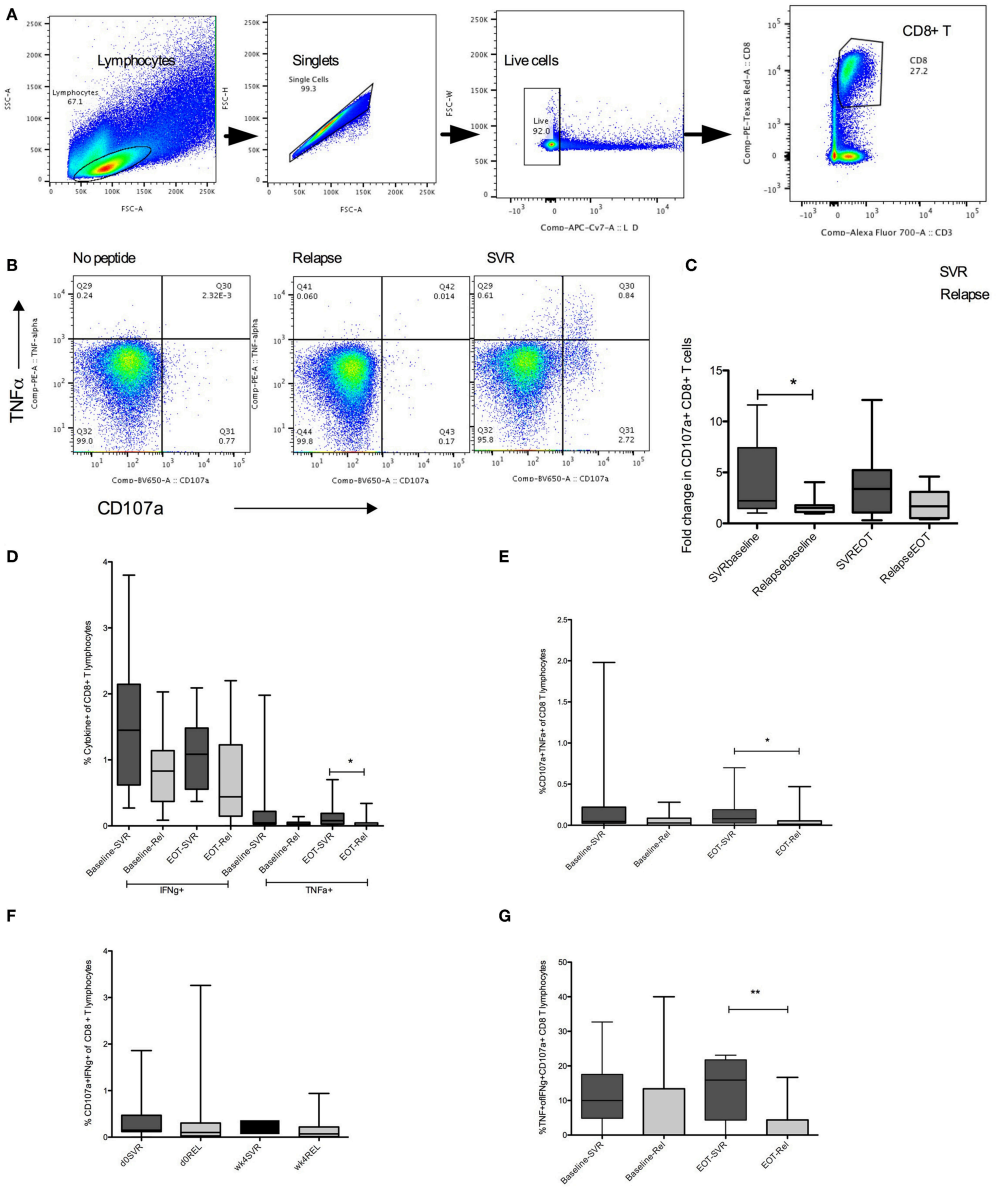
HCV specific cytokine response in SVR and Relapse groups. **(A)** Gating strategy to identify live, single CD8+ T lymphocytes. **(B)** CD107a and TNFα detected by intracellular cytokine staining in unstimulated or HCV pooled peptide stimulated cultures in representative relapse and SVR sample. **(C)** Group mean values for fold change in HCV specific CD107a+ expression in CD8+ T cells calculated as ratio of CD107a+ CD8 T cells in HCV peptide stimulated to unstimulated wells at baseline (d0) and end of treatment (EOT) **(D–G)** Frequencies of HCV specific double or triple cytokine producing CD8+ T cells from SVR or relapse groups at baseline (d0) and end of treatment (EOT). Statistical significances determined by Wilcoxon matched paired test for within group, Mann Whitney test for between group comparisons, *p* ≤ 0.05 is significant. D0, baseline; wk4, EOT, Rel, relpase. *p ≤ 0.05, **p ≤ 0.01.

Defects in HCV specific CD8 T cell cytotoxic capacity and cytokine production are associated with viral persistence (14, 15). We hypothesized that upon viral suppression with DAA, patients with stronger HCV specific immunity will be able to control virus replication when treatment ends at 4 weeks. To test for HCV specific cytokine production (IFNγ, TNFα, IL-2) and degranulation (CD107a), peripheral blood mononuclear cells from patients were stimulated with overlapping HCV peptides. Increased cytotoxic capacity determined by CD107a expression in CD8^+^ T cells in response to HCV peptide stimulation was observed at baseline in patients that achieved SVR (mean SVR vs. relapse fold CD107a, 1.36 vs. 0.3) (Figure 3). Analysis of HCV specific cytokine production showed higher TNFα production at EOT from SVR group compared to those who relapsed (mean SVR vs. relapse, 0.13 vs. 0.04%) (Figure 3). Higher frequencies of multiple cytokine producing CD8^+^ T cells: CD107a^+^TNFα^+^ (mean SVR vs. relapse, 0.13 vs. 0.05%) and %TNFα^+^IFNγ^+^CD107a^+^ (mean SVR vs. relapse, 13.3 vs. 3.4%) were present in SVR group at EOT than relapsers. Thus, patients who responded to 4 weeks therapy had higher HCV specific cytokine response at end of therapy. Improved proliferation of HCV specific CD8^+^ T cells within 4 weeks of treatment initiation among those that achieved SVR was reported previously (12). Higher frequencies of HCV specific CTLs at baseline that persisted throughout therapy in SVR group may aid in virus clearance in this group of patients.

### PD-1+CD8^+^ T Cells Co-expressing Inhibitory Receptors Contain HCV Specific Functional CD8^+^ T Cells

**Figure 4:**
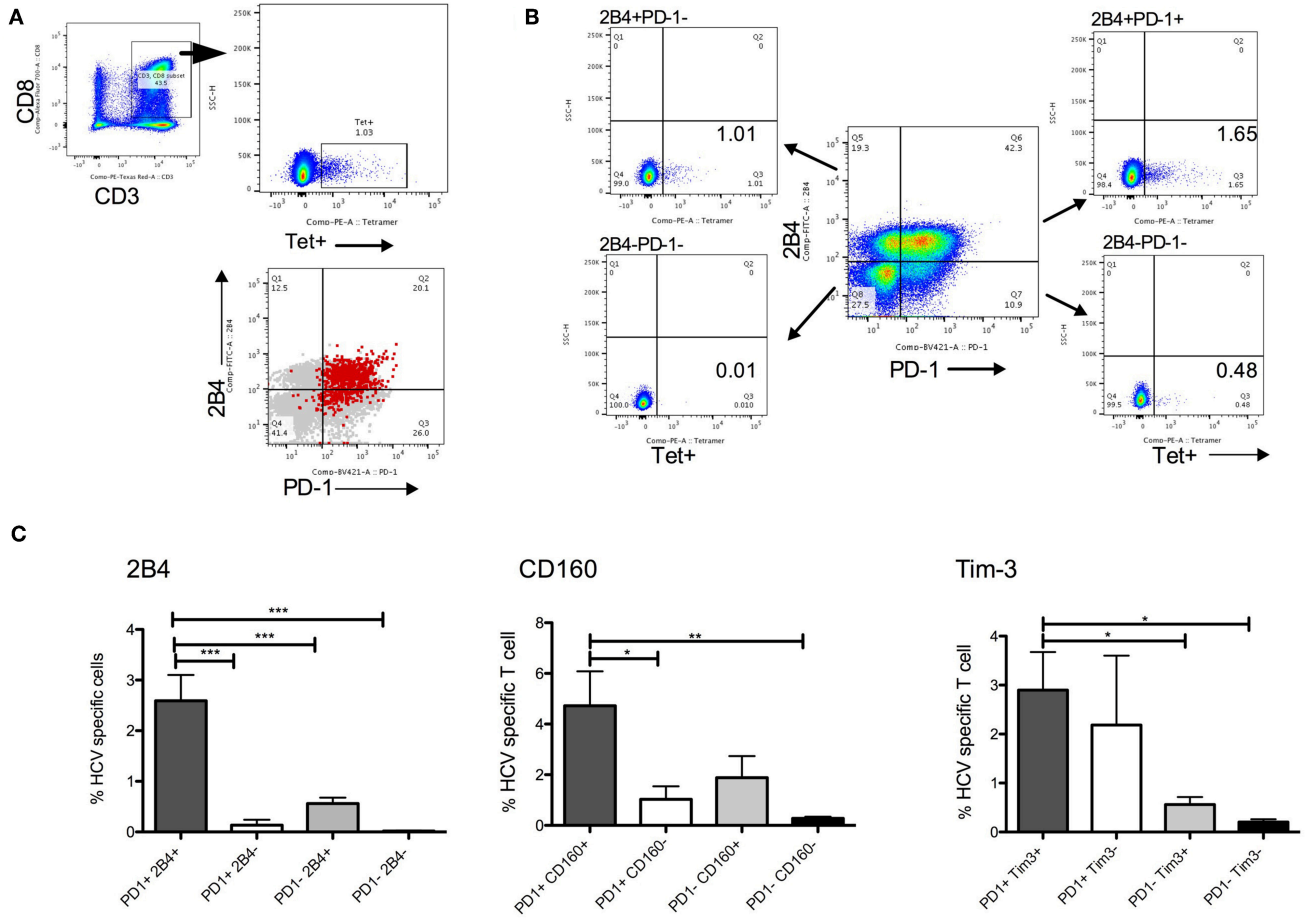
HCV specific CD8+ T cells in PD-1+ CD8 T cell subsets. **(A)** Gating strategy to detect HCV tetramer (tet+) CD8+ T cells. PD-1 and 2B4 expression among global (gray) and HCV tet+ (red) CD8+ T cells show most tet+ cells in PD-1+2B4+ subset. **(B)** Example of CD8 T cell subsets distinguished by PD-1 and 2B4 expression (2B4+PD-1-, 2B4-PD-1-, 2B4-PD-1+, 2B4+PD-1+) show relative abundance of HCV tet+ cells in four subsets. **(C)** Frequency of HCV tet+ T cells in CD8 T cells subsets distinguished by PD-1/2B4, PD-1/CD160 and PD-1/Tim-3 show average tet+ cells in different subsets (*N* = 5 HCV HLA-A*02 patients). Statistical significances determined by 1 Way ANOVA with Turkey's Multiple Comparison Test, *p ≤ 0.05, **p ≤ 0.01, ***p ≤ 0.001.

PD-1 is a well-established inhibitory receptor, expression of which on antigen specific T cells associates with immune dysfunction in chronic HCV (8, 9). Higher expression of PD-1 and other co-inhibitory receptors as predictors for SVR is therefore counterintuitive. However, since PD-1 and other inhibitory receptors are upregulated during chronic infection due to constant antigenic receptor stimulation, it is reasonable to expect that virus specific T cells will have higher expression of these receptors or are contained within these subsets. We performed immunophenotyping using HCV tetramers in samples from 5 HLA-A^*^02 HCV patients. HCV tetramer positive (tet^+^) CD8^+^ T cells were present within PD-1^+^ subsets that co-expressed other IRs, with significantly higher tet^+^ cells detected in PD-1^+^2B4^+^, PD-1^+^CD160^+^, or PD-1^+^Tim-3^+^ compared with single or no IR expressing subsets (Figure 4). While we have not evacuated tetramer positive cells frequency in all patients in SVR and relapse groups due to unavailability of corresponding MHC class I tetramers, based on our results, higher baseline frequencies of PD-1^+^Tet^+^ T cells will also likely associate with ability to achieve SVR with such short duration therapy.

**Figure 5:**
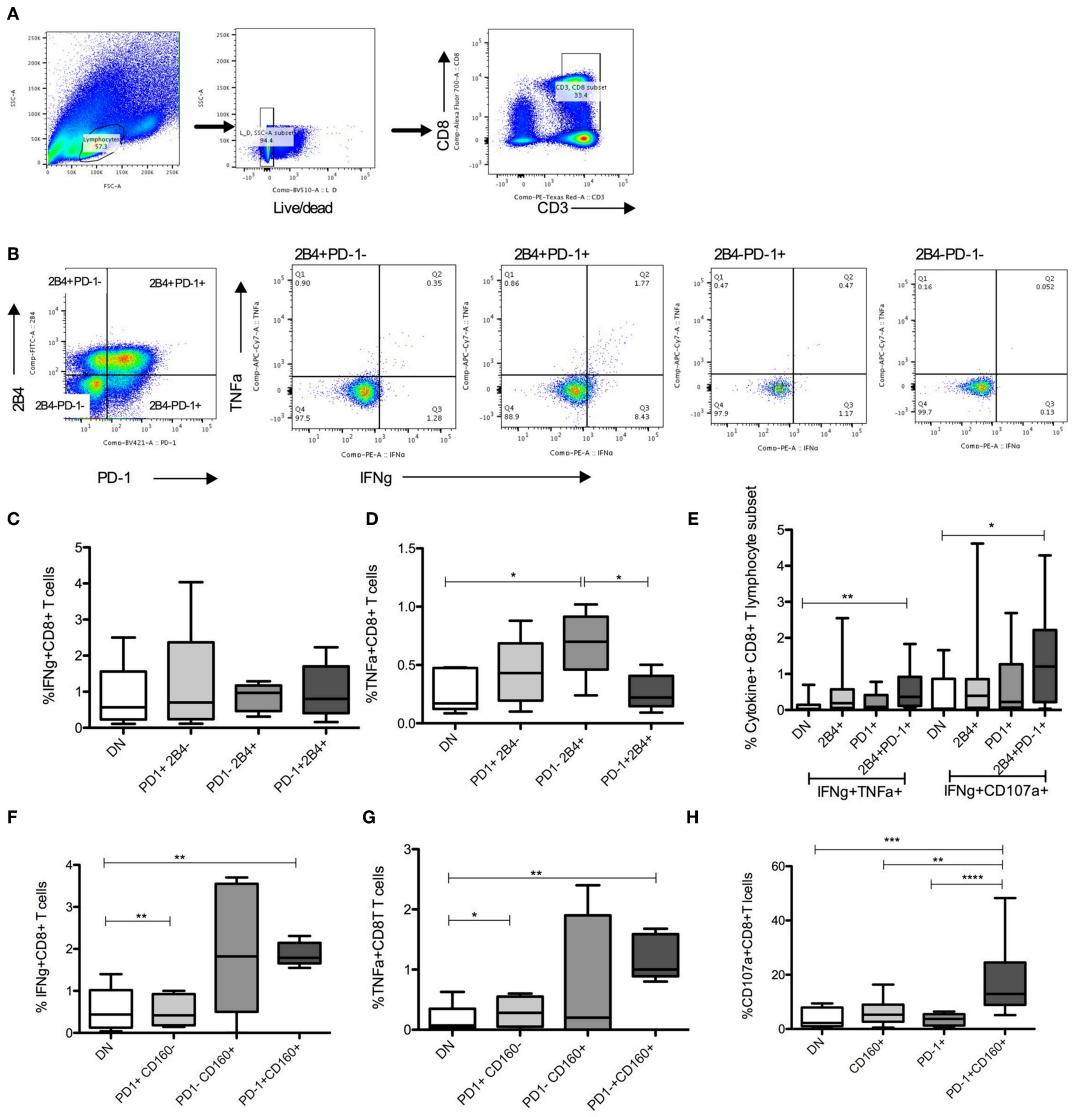
HCV specific cytokines in PD-1+ CD8 T lymphocyte subsets. **(A,B)** Gating strategy to identify live CD8+ lymphocyte subsets identified by PD-1 and 2B4 expression. **(C–E)** Frequencies of HCV specific cytokine (IFNγ+, TNFα+, CD107a+) producing cells among 4 distinct subsets of CD8+ T cells based on PD-1 and 2B4 expression (DN: PD-1-2B4-; 2B4+, PD-1+, and 2B4+PD-1+). **(F–H)** Frequencies of cytokine positive cells among 4 subsets of CD8+ T cells distinguished based on PD-1 and CD160 expression (DN: PD-1-CD160-; CD160+, PD-1+, and PD-1+CD160+) in response to HCV peptide stimulation. Statistical significances determined by 1 Way ANOVA with Turkey's Multiple Comparison Test, *p ≤ 0.05, **p ≤ 0.01, ***p ≤ 0.001, ****p ≤ 0.0001.

To test the functional potential of CD8^+^ T cell subsets that were identified in prediction model, we compared frequencies of cytokine producing cells in subsets defined by IRs expression. Highest levels of HCV specific TNFα^+^ and multiple cytokine (IFNγ^+^TNFα^+^ and IFNγ^+^CD107a^+^) expression was present in PD-1^+^2B4^+^ compared with single or no IR expressing CD8^+^ T lymphocyte subsets (Figures 5A–E). Similarly, PD-1^+^CD160^+^ CD8^+^ T expressed higher levels of HCV specific IFNγ, TNFα, and CD107a compared with single or no IR expressing subsets (Figures 5F–H).

Thus, HCV specific CD8^+^ T cells are present in PD-1 expressing subsets and these subsets have anti-HCV functional capacity.

## Discussion

In this study, we show that HCV specific immunity associates with achieving sustained virologic response in chronic HCV patients treated with direct acting antivirals for short duration (4 weeks). We demonstrate that higher frequencies of CD8^+^ T lymphocytes expressing PD-1 along with other inhibitory receptors 2B4, CD160, and Tim-3, are present at baseline and at end of therapy in patients that eventually achieve SVR. Successful prediction of ~80% SVR achievers can be made using frequencies of peripheral PD-1^+^ T cell subsets at the time of treatment initiation. Further, we show that these subsets contain HCV tetramer positive cells and possess HCV specific functional potential, thus providing basis for their association with achieving SVR.

Combination direct acting antiviral therapies of 8–12 weeks are highly effective for treatment of chronic HCV infection. To improve overall treatment adherence and costs associated with these therapies, shortening of therapies to <8 weeks has been explored with a few regimens (16). A personalized approach based on predictors of success or failure with short term treatment will be central to implementation of regimens shorter than 8–12 weeks. We previously reported factors predicting response to short duration therapy include lower viral load at baseline, young age, HCV genotype 1b infection, and absence of RAVs that confer more than 20 times resistance to therapy (3). What role, if any, host immunity plays in achieving SVR with DAA however is not clear. Natural resolution of acute HCV infection depends on HCV specific CD4^+^ T cell response (17–19), whereas a poor CD8^+^ T cells in early stage may increase likelihood of chronic viral persistence (8, 20). Selection of high-avidity CD8 T cells correlated with control of primary infection (21, 22). Chimpanzees studies allowed the mapping of specific T cell responses during chronic infection with CD8 T cells being linked to HCV control (23, 24). The evidence that host immunity can control HCV re-infection comes from infected untreated patients who spontaneously clear the infection (25). Furthermore, CD8^+^ T cell response against a broad range of HCV epitopes were observed among patients who spontaneously cleared HCV infection but not in those with persistent infection (26, 27). Additionally, protection from viral persistence during re-infection with HCV is dependent upon focused CD8 repertoire comprised of high avidity polyfunctional cells (28).

Unlike for natural infection outcomes, immune system's role in virus control during antiviral treatment is not clear. For IFN-a-based therapy chronic innate immune activation negatively associates with response to therapy (7). Higher levels of pretreatment intrahepatic interferon stimulated genes (ISG) expression and activated NK cells negatively correlate with subsequent virologic treatment response (29) arguing that in already activated immune system immune enhancement with interferon treatment is incremental. Treatment outcome with interferon based treatment was not associated with CD8^+^ T cell response (30), however HCV specific CD8^+^ T-cells at 12 weeks after treatment with PEG-IFN/ribavirin correlated with SVR (31). Role of immunity in achieving SVR with treatment regimens utilizing direct antivirals is not clear. By using combination antiviral agents these therapies act on various steps of vial life cycle and prevent naturally occurring resistant variants, however whether virus specific immunity is part of mechanism for achieving SVR is debated. Recovery of HCV specific CTL response accompanied by downregulation of inhibitory receptor expression on virus specific T cells in only those who achieve SVR with DAA therapy occurs (12). We also showed improved HCV specific immunity in those who achieved SVR with retreatment (13). Thus, immune dysfunction partly recovers in those who achieve SVR. To answer whether pretreatment differences in HCV specific immunity associate with a successful response to DAA, we took the approach of looking at a regimen that worked in a small subset of treated patients hypothesizing host immunity was a determining factor in success or failure. The finding of higher frequencies of HCV specific CD8^+^ T cells with cytotoxic capacity in SVR group agrees with a role for these cells described in resolving infection. The unexpected observation was higher frequencies of global PD-1^+^CD8^+^ T cells that co-express inhibitory receptors in responders. Expression of inhibitory receptors is regarded as hallmark of functional immune exhaustion during chronic infections and cancer. Higher HCV specific T cell immune exhaustion is present in relapsers to DAA treatment (12). HCV specific exhausted cells are of heterogeneous nature, and their survival upon DAA mediated antigen removal varies based on stage of exhaustion. The TCF1+CD127+PD1+ HCV specific CD8^+^ T cells exist in chronic patient during and long time after HCV elimination, with features of antigen independent survival and recall proliferation, whereas terminally exhausted CD127-PD-1hi CD8^+^ T cells were eliminated upon antigen removal (9). We observed higher levels of PD-1 expressing T cell subsets in patients that achieved SVR, both at baseline and at EOT compared to patients in relapse group; however at week 16 (12 weeks after EOT), significant reduction in these substes was present in SVR group only. This possibly reflects the elimination of antigen specific cells within these subsets after successful antigen removal in this group. PD-1 and other inhibitory receptor expression increases due to continuous signaling of TCR by viral antigens and antigen specific cells are expected to express multiple inhibitory receptors. HIV specific CD8^+^ T cells are predominantly PD-1^+^CD160^+^ (32). In chronically SIV infected macaques, PD-1^+^ subset does not loose proliferative capacity and responds with CD107a, IFNγ, and TNFα production to antigenic and SEB stimulation (33). In breast cancer infiltrating CD8^+^ T cells, robust proliferation, degranulation and cytokine producing capacity is present despite high expression of PD-1 (34).

For hepatitis B, antigen specific functional CD8^+^ T cells are exclusively present in the PD-1^+^ subset (35), and chronic patients with superior antiviral function upon therapy interruption had higher expression of PD-1 on their global CD8^+^ T cells. Isolated PD-1^+^ (and not PD-1^−^) T cells had HBV specific cytokine (IFNγ) response and this function correlated with absence of HBV flare upon therapy discontinuation (35). Example of chronic LCMV infection of mouse demonstrated that PD-1^+^ cells are virus specific and expression of PD-1 preserves antigen specific cells from overstimulation, excessive proliferation and terminal differentiation (36). Thus, PD-1 is associated with long-term persistence of virus specific T cells in human virus infections and potentially has a beneficial role in preservation of these cells. With this rationale, in our cohorts of patients treated with short term therapy, those with higher levels of inhibitory molecules on T cells would have better preservation of antigen specific cells. Upon virus suppression achieved with antivirals, these cells then are able to control residual virus at end of therapy. Overall, our results indicate that host antiviral immunity is associated with achieving SVR with short duration DAA therapy. This is an important step in our understanding of role of antiviral immunity in influencing outcome with direct acting antiviral therapy for HCV. So far, the clinical biomarkers that predict SVR to ultra-short duration treatment included viral factors such as viral load, genotype 1b and lack of resistant mutants. Results presented here show that host immune subsets with antiviral potential are also associated with enhanced viral clearance.

## Data Availability

The raw data supporting the conclusions of this manuscript will be made available by the authors, without undue reservation, to any qualified researcher.

## Ethics Statement

All participants signed informed consent approved by the National Institute of Allergy and Infectious Disease Institutional Review Board at the time of screening and enrollment and all samples were anonymized. All methods utilized for this study were performed in accordance with the relevant guidelines and regulations.

## Author Contributions

SR performed experiments and analyzed data. KS performed statistical analysis and edited the manuscript. AN provided clinical expertise and samples. SB edited manuscript for intellectual content. SK participated in study design and manuscript editing, provided clinical expertise, and financial support. BP designed the study, drafted the manuscript and provided funding, and overall project direction.

### Conflict of Interest Statement

The authors declare that the research was conducted in the absence of any commercial or financial relationships that could be construed as a potential conflict of interest.
