# Supplementary material for: Peripheral PD-1+ T Cells Co-expressing Inhibitory Receptors Predict SVR With Ultra Short Duration DAA Therapy in HCV Infection
Source: Front Immunol. 2019 Jun 27;10:1470. doi: 10.3389/fimmu.2019.01470 (PMC6610534; doi:10.3389/fimmu.2019.01470)
Supplement: Supplementary file 1 [file Table_1.docx]

| CD8 Markers at Baseline | Frequency in SVR | Frequency in Relapse | P value |
| --- | --- | --- | --- |
| CD160+, PD-1^+^ | 1.4 ± 1.1 | 0.6 ± 0.5 | 0.02 |
| Tim-3+, PD-1^+^ | 1.6 ± 0.8 | 0.8 ± 0.7 | 0.01 |
| CTLA-4^+^, PD-1^-^ | 2 ± 0.8 | 5.8 ± 2.2 | <0.0001 |
| CD8 Markers at EOT | Frequency in SVR | Frequency in Relapse | P value |
| PD1 hi | 24.2 ± 9.4 | 16.9 ±9.0 | 0.04 |
| 2B4^+^, PD-1^+^ | 17.1 ± 7.3 | 11.0 ± 7.7 | 0.04 |
| KLRG-1^+^, PD-1^+^ | 18.9 ± 8.9 | 12.3 ± 5.7 | 0.03 |
| CTLA-4^+^, PD-1^-^ | 1.8 ± 0.8 | 5.7 ± 2 | <0.0001 |
| Blimp^+^, PD-1^+^ | 10.7 ± 5.3 | 6.7 ± 4.2 | 0.04 |
| CD8 Markers at Wk16 | Frequency in SVR | Frequency in Relapse | P value |
| Eomes^+^ T-bet^-^  (+) | 21.2 ± 11.0 | 24.0 ± 9.3 | 0.02 |
| CCR7^-^ CD45RO^+^ | 24.9 ± 8.8 | 16 ± 7.6 | 0.01 |
|  |  |  |  |
| CD4 Markers at Baseline | Frequency in SVR | Frequency in Relapse | P value |
| CD160^+^, PD-1^+^ | 2.1 ± 1.6 | 0.9 ± 0.6 | 0.03 |
| CTLA-4^+^, PD-1^-^ | 4.8 ± 1.8 | 7.6 ± 2.6 | 0.004 |
| Eomes^+^ T-bet^-^  PD-1^+^ | 9.7 ± 3.2 | 6.5 ± 2.6 | 0.008 |
| Eomes^-^ T-bet^+^ PD-1^+^ | 2.2 ± 3.1 | 6.6 ± 6.8 | 0.01 |
| Blimp^+^, PD-1^+^ | 16.2 ± 8.4 | 10.5 ± 5.4 | 0.04 |
| CD4 Markers at EOT | Frequency in SVR | Frequency in Relapse | P value |
| CTLA-4^+^, PD-1^-^ | 4.7 ± 2.1 | 7.5 ± 2.4 | 0.006 |
| Eomes^-^ T-bet^+^ PD-1^+^ | 1.7 ± 2.4 | 6.1± 6.0 | 0.008 |
| Blimp^+^, PD-1^+^ | 15.8 ± 6.4 | 10.5 ± 6.1 | 0.03 |
| PD1+, CD39+ | 3.9 ± 1.5 | 2.4 ± 1.3 | 0.01 |
| CD4 Markers at Wk16 | Frequency in SVR | Frequency in Relapse | P value |
| 2B4^+^, PD-1^+^ | 2.9 ± 3.0 | 7.0 ± 5.7 | 0.02 |
| CCR7^-^ CD45RO^+^ | 36.5 ± 13 | 24.3 ± 8 | 0.01 |

**Supplementary Table 1: CD8+ and CD4+ T cell markers differentially expressed between SVR and relapse groups at baseline, EOT and week 16.**
