# Supplementary material for: Peripheral PD-1+ T Cells Co-expressing Inhibitory Receptors Predict SVR With Ultra Short Duration DAA Therapy in HCV Infection
Source: Front Immunol. 2019 Jun 27;10:1470. doi: 10.3389/fimmu.2019.01470 (PMC6610534; doi:10.3389/fimmu.2019.01470)
Supplement: Supplementary file 2 [file Data_Sheet_1.PDF]

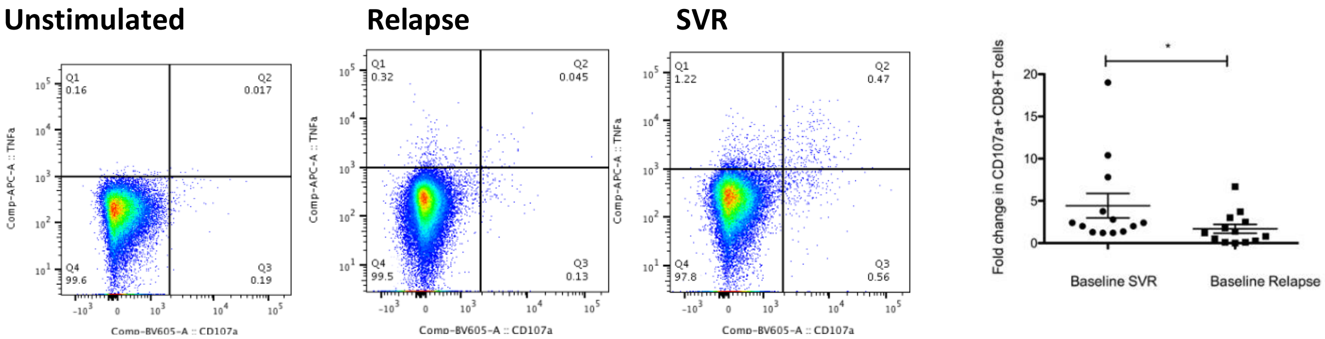

Cytokine response to HCV core peptide stimulation in Relapse or SVR patients
